# Dependence of DCE-MRI biomarker values on analysis algorithm

**DOI:** 10.1371/journal.pone.0130168

**Published:** 2015-07-24

**Authors:** Chaan S. Ng, Wei Wei, James A. Bankson, Murali K. Ravoori, Lin Han, David W. Brammer, Sherry Klumpp, John C. Waterton, Edward F. Jackson

**Affiliations:** 1 Department of Radiology, University of Texas M.D. Anderson Cancer Center, Houston, Texas, United States of America; 2 Department of Biostatistics, University of Texas M.D. Anderson Cancer Center, Houston, Texas, United States of America; 3 Department of Biostatistics Imaging Physics, University of Texas M.D. Anderson Cancer Center, Houston, Texas, United States of America; 4 Department of Biostatistics Veterinary Medicine and Surgery, University of Texas M.D. Anderson Cancer Center, Houston, Texas, United States of America; 5 Personalised Healthcare and Biomarkers, AstraZeneca, Alderley Park, Cheshire, United Kingdom; 6 Department of Medical Physics, University of Wisconsin, Madison, WI, United States of America; Northwestern University Feinberg School of Medicine, UNITED STATES

## Abstract

**Background:**

Dynamic contrast-enhanced MRI (DCE-MRI) biomarkers have proven utility in tumors in evaluating microvascular perfusion and permeability, but it is unclear whether measurements made in different centers are comparable due to methodological differences.

**Purpose:**

To evaluate how commonly utilized analytical methods for DCE-MRI biomarkers affect both the absolute parameter values and repeatability.

**Materials and Methods:**

DCE-MRI was performed on three consecutive days in twelve rats bearing C6 xenografts. Endothelial transfer constant (*K* ^trans^), extracellular extravascular space volume fraction (*v*_e_), and contrast agent reflux rate constant (*k*_ep_) measures were computed using: 2-parameter (“Tofts” or “standard Kety”) *vs*. 3-parameter (“General Kinetic” or “extended Kety”) compartmental models (including blood plasma volume fraction (*v*_p_) with 3-parameter models); individual- *vs*. population-based vascular input functions (VIFs); and pixel-by-pixel *vs*. whole tumor-ROI. Variability was evaluated by within-subject coefficient of variation (wCV) and variance components analyses.

**Results:**

DCE-MRI absolute parameter values and wCVs varied widely by analytical method. Absolute parameter values ranged, as follows, median *K* ^trans^, 0.09–0.18 min^-1^; *k*_ep_, 0.51–0.92 min^-1^; *v*_e_, 0.17–0.23; and *v*_p_, 0.02–0.04. wCVs also varied widely by analytical method, as follows: mean *K* ^trans^, 32.9–61.9%; *k*_ep_, 11.6–41.9%; *v*_e_, 16.1–54.9%; and *v*_p_, 53.9–77.2%. *K* ^trans^ and *k*_ep_ values were lower with 3- than 2-parameter modeling (p<0.0001); *k*_ep_ and *v*_p_ were lower with pixel- than whole-ROI analyses (p<0.0006). wCVs were significantly smaller for *v*_e_, and larger for *k*_ep_, with individual- than population-based VIFs.

**Conclusions:**

DCE-MRI parameter values and repeatability can vary widely by analytical methodology. Absolute values of DCE-MRI biomarkers are unlikely to be comparable between different studies unless analyses are carefully standardized.

## Introduction

Imaging biomarkers can assess tumor perfusion and permeability, and are useful in assessing response to therapy [1]. In particular, dynamic contrast-enhanced magnetic resonance imaging (DCE-MRI) provides biomarkers of tissue perfusion with proven utility in oncologic imaging, including the assessment of treatment responses and development of anti-cancer therapies [2–4]. However, these biomarkers are little-used outside the single-center setting, probably because different implementations of the imaging acquisition and analysis have not been shown to provide comparable biomarker values.

The DCE-MRI technique has been, and can be, used in clinical and pre-clinical settings, the latter in particular where novel therapeutic agents are under investigation [5–9]. In both settings, quantitative evaluations of the changes in derived tissue perfusion biomarkers have often been the main objectives. While any one study will use the same algorithm and analytical implementation for all subjects pre- and post-therapy, there is little consistency between studies. Although biomarker values are quoted in absolute units (e.g. k^trans^ /min^-1^), it is unclear to what extent absolute values reported from different studies are comparable. In this study we evaluated three important analysis options: the choice of model, the method of derivation of the input function, and the algorithm for aggregating pixel-wise data to derive whole-tumor biomarkers.

The technique of DCE-MRI depends on acquiring dynamic MRI data and applying an appropriate physiological model to that data. A variety of tracer kinetic models have been developed for these purposes; two commonly utilized models are variably termed the Tofts and Kermode, “standard” Kety, or 2-parameter model [10–12], and the generalized kinetic, “extended” Kety, or 3-parameter model [13]. Application of these models allows derivation of specific MRI perfusion parameters, such as the endothelial transfer constant (*K* ^trans^), the contrast agent reflux rate constant (*k*_ep_), the extracellular extravascular space volume fraction (*v*_e_), and the blood plasma volume fraction (*v*_p_).

Model-based derivations of DCE-MRI parameters require a vascular input function (VIF). Obtaining reliable VIF data has been, and is, challenging, particularly in pre-clinical settings where even the central vessels, *e*.*g*., aorta and inferior vena cava, are extremely small. Imaging artifacts and the high cardiac rate of small animals add to the challenges. The unreliable nature of some VIFs from individual subjects can potentially confound the overall estimates of perfusion parameter values. In these situations, model or population-based VIFs have been suggested [10,14–20].

Tissue perfusion parameters for a region of interest (ROI) can be derived on a “whole tumor” or “pixel-by-pixel” basis. Pixel-level data in principle offers a more detailed evaluation and allows for intratumoral assessment of the heterogeneity of each measured parameter [20]. It is, however, prone to the potential challenges of additional computation time and signal-to-noise ratio limitations.

In this study, we computed DCE-MRI parameter values utilizing all combinations of the above methods on DCE-MRI images obtained on three successive days in each of twelve rat xenografts. Absolute parameter values and repeatability were compared. An understanding of repeatability provides data for assessing study results and for study design (namely, determining sample sizes).

Our objectives were to compare the absolute values and test-retest repeatability of DCE-MRI parameters analyzed by two tracer kinetic models (2-parameter *vs*. 3-parameter), two different VIF input strategies (individual- vs. population-based), and two tissue ROI approaches (whole tumor *vs*. pixel-by-pixel) in a rat tumor model.

## Materials and Methods

The study was approved by the Institutional Animal Care and Use Committee (IACUC) of The University of Texas M.D. Anderson Cancer Center (Protocol Number 09-06-12041). C6 rat glioma cells were obtained from the American Type Culture Collection (Manassas, VA, USA). Five thousand C6 cells were injected subcutaneously into male Crl: NIH-*Foxn1* ^rnu^ T-cell deficient, athymic nude rats (Charles River, Wilmington, MA). Rats weighted approximately 220 grams at the time of the experiment. Cells were injected in the flank region, at the approximate axial level of the inferior aspect of the kidneys and distal aorta. Tumor measurements were undertaken using calipers, and tumors allowed to grow until they reached a nominal size of approximately 1 cm diameter. Each rat then underwent DCE-MRI on three consecutive days. Animals were scanned in batches of 5 to 6 animals per cohort.

Animals were placed in an MRI-compatible cradle in which a 5-cm hole had been cut into which the subcutaneous tumor could be located. Hair from around the tumor was shaved, and the region of the tumor was placed in a “bath” of ultrasound gel to minimize air/tumor susceptibility effects in the MRI imaging studies. A temperature controlled pad was placed underneath the animals, and the animals were gently immobilized with tape. Animals were anesthetized with 1–2% isoflurane in a 1 l/min O_2_ flow, and imaging was undertaken in free respiration throughout. The imaging volume was targeted on the central portion of the tumor.

An estimate of tumor volume (cm^3^) was obtained from the formula for a spheroid, *i*.*e*., (π*X*Y^2^)/6000, where X and Y (in mm) were orthogonal tumor diameter measurements.

Data from a total of 12 sets of three consecutive days of scanning were obtained: 10 rats underwent three consecutive DCE-MRI studies during a single week period; one animal underwent the three consecutive DCE-MRI studies in two separate weeks. There was a technical scanning failure on one MRI scan visit in one rat. The median size of tumors was 0.67 cm^3^ (range 0.09–1.53 cm^3^). At the end of the study, the rats were euthanized humanely by inhalation of carbon dioxide.

### DCE-MRI technique

MRI studies were undertaken using a 7.0 Tesla / 30 cm bore dedicated animal MRI scanner (Bruker BioSpin, Billerica, MA). The MR scanning protocol consisted of the acquisition of sagittal and axial T_2_-weighted images, axial T_1_-weighted images, axial DCE-MRI images, and post-Gd axial T_1_-weighted images. For the DCE-MRI acquisition, a 3D fast spoiled gradient echo sequence was used with TE = 1.7ms, TR = 10ms, 15° excitation pulse, 16-mm slab thickness (yielding eight 2-mm slices), 128 x 80 matrix, and 60mm x 50mm field of view. To reduce artifacts in the VIF from inflow effects, a spoiled hermite magnetization preparation pulse was applied to excite an 8-cm slab located 2 mm caudal to the DCE-MRI slice package [21]. The temporal resolution was 6.4s, with total scan time of 320s (50 x 6.4s). Contrast agent was administered after 10 baseline scans were acquired.

The MRI contrast agent was delivered through a tail vein as follows: 0.2 mmol/kg dose of gadopentetate dimeglumine (Magnevist, Bayer Healthcare Pharmaceuticals, Wayne, NJ), via an MR-compatible injection system (Harvard Apparatus, PHD 2000 Programmable, Plymouth Meeting, PA). For a 200-gram rat, for example, 200 μl of contrast media at 1:5 dilution of Magnevist:saline was administered over a period of 10 seconds. This was followed by a saline flush of the same volume and injection rate.

The acquired DCE-MRI data were analyzed using the Kinmod module (version 3.0) within the CineTool (version 8.2.1) environment (GE Healthcare, Waukesha, WI), utilizing two tracer kinetic models (2-parameter *vs*. 3-parameter), two VIF input strategies (population-based *vs*. individual-based), and two ROI approaches (whole tumor cross-sectional ROI *vs*. pixel-by-pixel within the ROI).

### Tracer kinetic models: two- vs. three- parameter

Analyses were undertaken using two standard two-compartment tracer kinetic models: a) 2-parameter (“Tofts” or “standard Kety”) and b) 3-parameter (“General Kinetic” or “extended Kety”) model.

#### a) 2-parameter model:

$$C_{t}\left(t\right)=K^{trans}\;\int_{0}^{t}C_{p}\left(t\prime \right)e^{-\left[\frac{K^{trans}\left(t-t\prime \right)}{v_{e}}\right]}\;dt\prime$$

where *C*_t_(*t*) is the tracer concentration in tissue, *C*_p_(*t*) is the tracer concentration in arterial blood plasma, *K* ^trans^ is the volume endothelial transfer constant between blood plasma and extravascular extracellular space (in min^-1^), and *v*_e_ is the extravascular extracellular tissue volume fraction (dimensionless).

This model is variably named the Tofts and Kermode, Larsson, or “standard (flow-limited)” Kety model [10–12].

#### b) 3-parameter model:

$$C_{t}\left(t\right)=v_{p}C_{p}\left(t\right)+K^{trans}\;\int_{0}^{t}C_{p}\left(t\prime \right)e^{-\left[\frac{K^{trans}\left(t-t\prime \right)}{v_{e}}\right]}\;dt\prime$$

where the additional term, *v*_p_, is the blood plasma volume fraction (dimensionless).

This model is otherwise named the General Kinetic Model (GKM), or “extended” Kety model [12].

For both models, the contrast agent reflux rate constant, *k*_ep_ (in min^-1^) = *K* ^trans^ / *v*_e_.

### Vascular input function: individual vs. population

We evaluated two commonly utilized vascular input functions (VIF): a) individually measured, and b) population-based.

#### a) Individually measured VIF

**Fig 1:**
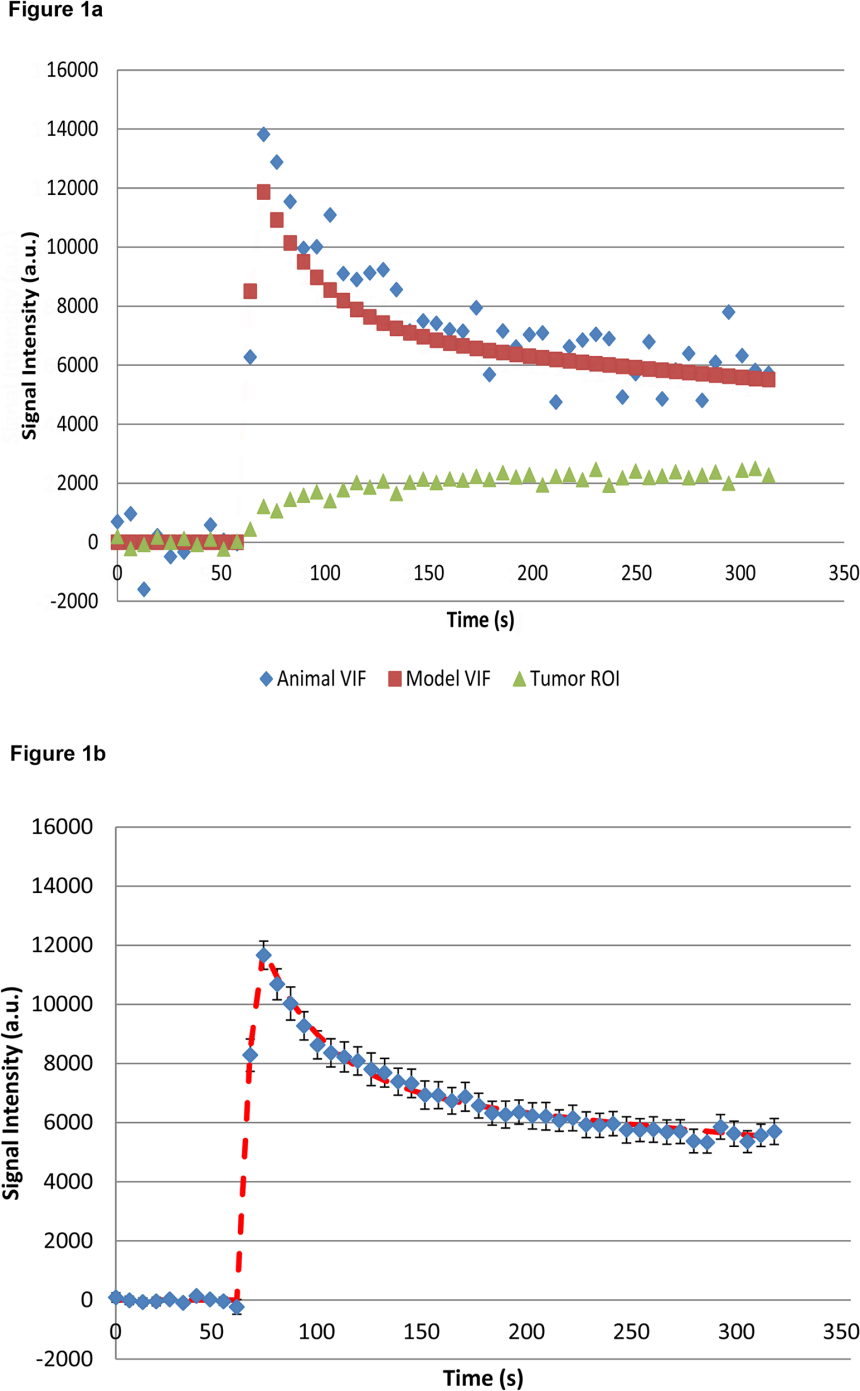
Example of signal intensity profiles. A) Vascular input function (VIF) from a representative individual animal (blue), and population average (red). Green symbols: tumor whole ROI data. Y-axis in units of signal intensity change from baseline, *ΔSI*. B) Average signal intensity change (VIF) from all animals and scan visits, including the standard error, for each data point (blue); and the fitted population-average VIF curve (red line). Y-axis in units of signal intensity change from baseline, *ΔSI*.

**Fig 2:**
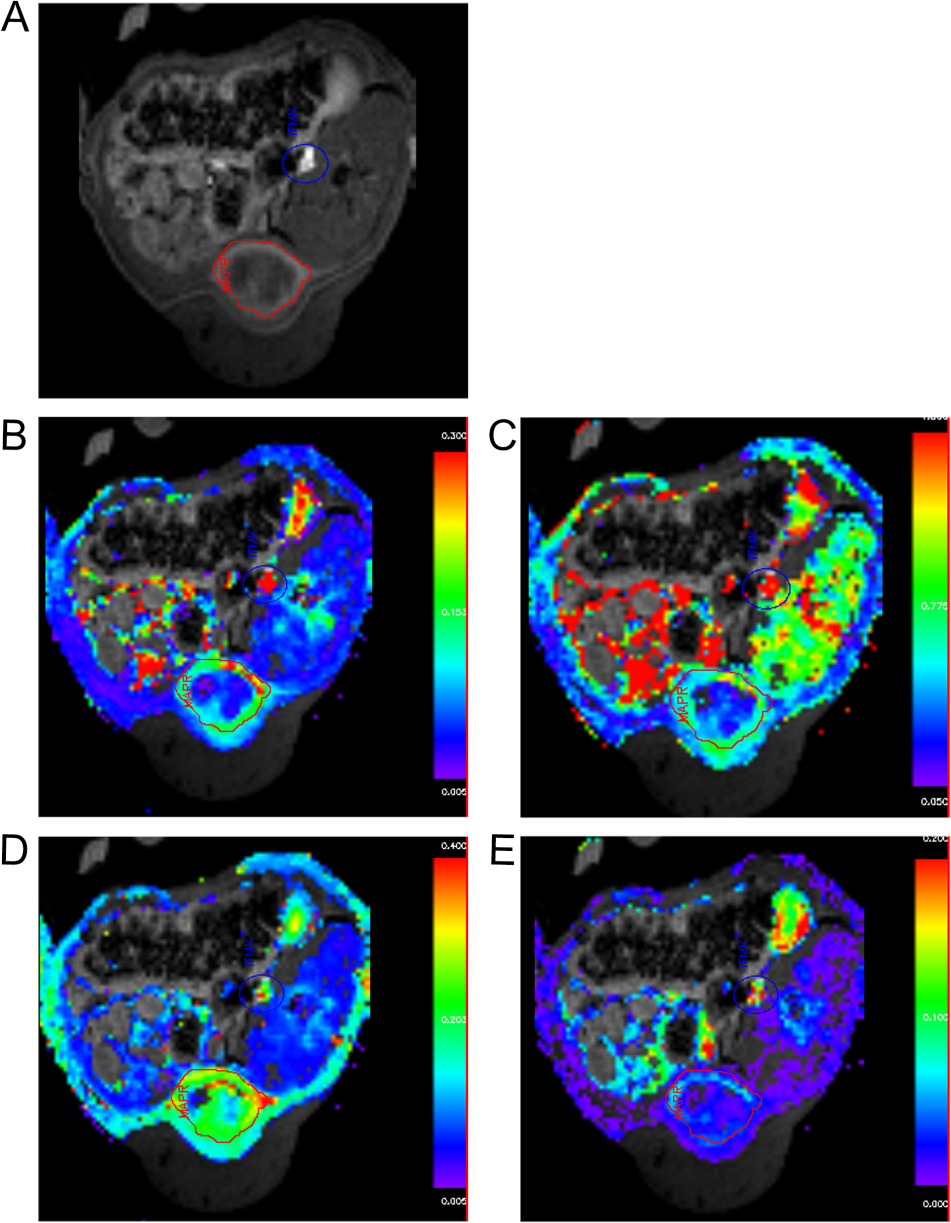
Illustrative parametric maps using 3-parameter physiological model, individual VIF and pixel based analysis. Same animal as Fig 1. A) Source trans-axial DCE-MRI image [blue outline is VIF input mask; red outline is tumor ROI mask]; B) *K* ^trans^, C) *k*_ep_, D) *v*_e_, and E) *v*_p_ parameteric maps.

For each animal and time point, the VIF was obtained using a mask ROI defined by the study radiologist (CSN, more than 15 years’ experience) containing the inferior vena cava in an imaging section near the center of the DCE-MRI scan volume. From the mask ROI, the VIF was determined using an automated VIF identification algorithm within the Kinmod module (Figs 1A and 2A). The VIF represents vascular gadopentetate concentration measured in units of signal intensity change from baseline as a function of time, *i*.*e*., *ΔSI*(*t*) = *SI*(*t*)–*SI*(*baseline*), where *SI*(*baseline*) was obtained by averaging the signal intensity from frames 5–10, after achieving steady state but prior to contrast agent administration [22].

#### b) Population average VIF

The population-based VIF was derived from the individual VIFs (discussed above) obtained from all 12 rats of the study cohort and all available scan visits, *i*.*e*., 3 visits per rat. The individual VIF curves for all animals were averaged and the resulting data fitted to a biexponential function resulting in a population VIF given by

$$C_{p}\left(t\right)=0.64\;e^{-0.0333t}+0.42\;e^{-0.0010t}$$

where *t* is in seconds and *C*_p_(*t*) is in units of signal intensity change, *ΔSI*(*t*) (Fig 1B). The short and long component amplitude and clearance rates thus determined were in good agreement with previously published data [23,24].

### Tumor ROI approaches: pixel-by-pixel vs. whole

ROIs were drawn by a single observer (CSN) using an electronic cursor and mouse for the three central slices of tumor for each rat. DCE-MRI parameters were computed using all combinations of the two tracer kinetic models and the two VIF methods above, using the same set of ROIs for each tumor. DCE-MRI parameter values were obtained for each tumor ROI based on: a) whole cross section ROI data, and b) pixel-by-pixel data within the ROI. In both cases, the values of *C*_*t*_ *(t)* represent the tissue gadopentetate concentration measured in units of signal intensity change from baseline as a function of time, as for the VIF curves. The same ROIs for any given rat were used in each analysis.

#### a) “Pixel-by-pixel ROI” analyses

The intensity values of individual pixels within the ROIs were used in computations. Pixels which demonstrated non-significant changes in signal intensity following contrast agent injection, specifically less than a 50% increase, were not included in further statistical analyses. In addition, positive-definite limits were placed on all fit parameters and any pixels for which fit parameters were outside of such limits were flagged as having fit parameters that were “not a number” (NaN).

#### b) “Whole ROI” analyses

The average intensity value of the whole tumor ROI was used in computations.

The average of the DCE-MRI parameter values derived from each of the three imaging slices were used in subsequent analyses. Illustrative examples of DCE-MRI parametric maps are presented in Fig 2.

### Statistical analyses

Summary statistics of DCE-MRI parameters (*K* ^trans^, *k*_ep_, *v*_e_, and where applicable, *v*_p_) were provided in the form of median and range. All data were transformed to the logarithmic scale, due to right-skewness, prior to statistical analyses.

A linear mixed model was used to assess if each DCE-MRI parameter changed significantly over three days. The linear mixed model took into account the correlation between measurements of the same rat. No statistically significant trend was detected for any DCE-MRI parameter in the span of three days (results not shown).

Comparisons between DCE-MRI parameters by analytical method were also based on linear mixed models. Interactions between analytical methods and time were not significant (results not shown), therefore, analytical methods and time were fit as main effects only. All the pairwise comparisons between analytical methods were estimated and p-values were adjusted using the Bonferroni method to control the overall type I error rate at 5%.

A variance components analysis was used to estimate the inter- and intra-rat variances. The intra-rat coefficient of variation (wCV) was calculated using the Bland-Altman method [25] as follows: logarithms of the data were taken and within-rat standard deviations estimated, the results were back-transformed to the raw scale (anti-log), and a value of one was subtracted from them to obtain the wCVs.

All statistical analyses were two-sided and *p*-values of 0.05 or less were considered statistically significant. Statistical analysis was carried out using SAS version 9 (SAS Institute, Cary, NC). Plotting was performed using Spotfire S+ 8.2 (TIBCO Software Inc., Somerville, MA).

## Results

Summary statistics for the DCE-MRI parameters, by analytic method, are presented in Table 1. Overall, DCE-MRI parameter values varied widely across the various analytical methods, *i*.*e*. the tracer kinetic model, VIF input, and method of ROI analysis, with the range in median DCE-MRI parameter values as follows: *K* ^trans^, 0.09–0.18 min^-1^; *k*_ep_, 0.51–0.92 min^-1^; *v*_e_, 0.17–0.23; and *v*_p_, 0.02–0.04 (Table 1).

**Table 1:** Summary of DCE-MRI parameter values, by analytical method (n = 12). Model dependent (*K* ^trans^, *k*_ep_, *v*_e_ and *v*_p_).

				Whole ROI				Pixel ROI			
VIF	Parameter model	Parameter	Units	Median	IQR	Mean wCV	wCV bounds	Median	IQR	Mean wCV	wCV bounds
						%	%			%	%
**POP**	**2 para**	***K*** ^**trans**^	min^-1^	**0.17**	0.10–0.22	**57.8**	38.3–80.0	**0.17**	0.11–0.21	**50.1**	33.5–68.8
		***k***_**ep**_	min^-1^	**0.92**	0.83–1.03	14.6*	10.2–18.2	**0.78**	0.73–0.86	15.8*	11.0–20.9
		***v***_**e**_	unitless	**0.19**	0.12–0.27	**54.9** ^**††**^	36.5–75.8	**0.19**	0.14–0.27	**49.0** ^**††**^	32.8–67.2
**POP**	**3 para**	**K** ^**trans**^	min^-1^	**0.09**	0.07–0.14	**61.9**	40.9–86.2	**0.10**	0.07–0.14	**53.8**	35.8–74.1
		***k***_**ep**_	min^-1^	**0.59**	0.54–0.64	11.6*	8.11–15.2	**0.52**	0.48–0.56	14.1*	9.8–18.6
		***v***_**e**_	unitless	**0.17**	0.12–0.26	**53.7** ^**††**^	35.7–74.0	**0.18**	0.13–0.27	**52.1** ^**††**^	34.7–71.7
		***v***_**p**_	unitless	**0.04**	0.03–0.06	**54.5**	36.3–75.3	**0.04**	0.03–0.05	**53.9**	35.9–74.3
**INDIV**	**2 para**	***K*** ^**trans**^	min^-1^	**0.18**	0.15–0.25	**39.7**	26.8–53.8	**0.17**	0.14–0.23	**32.9**	22.4–44.3
		***k***_**ep**_	min^-1^	**0.85**	0.69–1.07	33.1**	22.6–44.6	**0.69**	0.58–0.92	34.3**	23.4–46.3
		***v***_**e**_	unitless	**0.22**	0.20–0.25	**16.1** ^**†**^	11.2–21.2	**0.23**	0.20–0.26	**16.9** ^**†**^	11.7–22.3
**INDIV**	**3 para**	***K*** ^**trans**^	min^-1^	**0.11**	0.09–0.14	**39.3**	26.6–53.3	**0.12**	0.10–0.14	**34.4**	23.4–46.4
		***k***_**ep**_	min^-1^	**0.56**	0.46–0.69	36.6**	24.9–49.5	**0.51**	0.39–0.62	41.9**	28.2–57.0
		***v***_**e**_	unitless	**0.21**	0.18–0.24	**16.4** ^**†**^	11.4–21.6	**0.23**	0.20–0.25	**18.7** ^**†**^	13.0–24.7
	** **	***v***_**p**_	unitless	**0.03**	0.02–0.06	**58.8**	39.0–81.5	**0.02**	0.01–0.04	**77.2**	50.2–109.02

POP: population averaged VIF

INDIV: individual measured VIF

IQR: inter-quartile range

wCV: within-subject coefficient of variation. Bounds: 95% lower- and upper-confidence limits

n/a: not applicable

* vs ** = significant differences

^**†**^ vs ^**††**^ = significant differences

The wCV values also varied widely by analytical method, with mean wCVs ranging as follows: *K* ^trans^, 32.9–61.9%; *k*_ep_, 11.6–41.9%; *v*_e_, 16.1–54.9%; and *v*_p_, 53.9–77.2% (Table 1).

### Effect of tracer kinetic model: 2-parameter vs. 3-parameter

**Fig 3:**
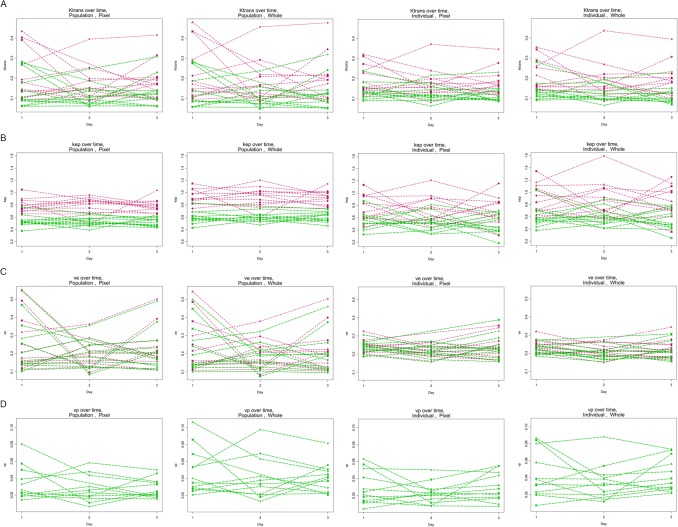
Scatter plots of 3 day time points, of horizontal row a) *K* ^trans^, b) *k*_ep_, c) *v*_e_, d) *v*_p_, by 2- (red lines) vs. 3-parameter (green lines) models; with separate plots for pixel-by-pixel vs. whole tumor analyses, and by individual- vs. population-based VIFs. Y-axes for *K* ^trans^ and *k*_ep_ in min^-1^: *v*_e_ and *v*_p_, unitless. Note: *v*_p_ can only be derived with the 3-parameter model. (Note: one missing data point for one rat)

*K* ^trans^ and *k*_ep_ were significantly and consistently lower when using the 3-parameter model compared to the 2-parameter model (p<0.0001); *v*_*e*_ values were not significantly different (Tables 1 and 2; Fig 3 (green vs. red datasets)).

**Table 2:** Summary of pairwise comparisons between DCE-MRI parameters by analytical method. P-values based on linear mixed model on the logarithmic scale. The linear mixed model took into account correlation between measurements from the same rat. A Bonferroni adjustment was used to control the overall type I error rate, which with 13 pairwise comparisons set the cutoff point for declaring statistical significance as 0.05/13 = 0.0038, which are bolded.

Parameter model	Comparison	Estimate	Standard Error	p-value
*K* ^trans^	2para *vs*. 3para model	0.449	0.053	**<0.0001**
*K* ^trans^	Individual *vs*. Population VIF	0.098	0.060	0.129
*K* ^trans^	Pixel *vs*. Whole ROI	0.002	0.047	0.966
*k*_ep_	2para *vs*. 3para model	0.419	0.021	**<0.0001**
*k*_ep_	Individual *vs*. Population VIF	-0.064	0.023	0.019
*k*_ep_	Pixel *vs*. Whole ROI	-0.145	0.021	**<0.0001**
*v*_e_	2para *vs*. 3para model	0.031	0.031	0.337
*v*_e_	Individual *vs*. Population VIF	0.175	0.051	0.006
*v*_e_	Pixel *vs*. Whole ROI	0.058	0.029	0.066
*v*_p_	Individual *vs*. Population VIF	-0.100	0.108	0.371
*v*_p_	Pixel *vs*. Whole ROI	-0.434	0.091	**0.0006**

The effects on repeatability of the DCE-MRI parameters (*K* ^trans^, *k*_ep_ and *v*_e_) were comparable, with no significant differences in wCVs across the two tracer kinetic models, as demonstrated by overlapping wCV confidence bounds for corresponding parameters (Table 1).

### Effect of VIF: individual- vs. population-based

There were no significant differences in DCE-MRI parameter values (*K* ^trans^, *k*_ep_, *v*_*e*_, and *v*_*p*_) when comparing the utilization of individual-based and population-based VIFs in analyses.

Utilization of individual-based VIFs was associated with significantly smaller wCVs for *v*_*e*_ than with the population-based VIF, and conversely for *k*_ep_ (Fig 3 (1^st^ and 2^nd^ vs. 3^rd^ and 4^th^ columns); Table 1, showing non-overlapping confidence bounds for corresponding wCVs). There were no significant differences for *K* ^trans^ and *v*_p_ when comparing individual- vs. population-based VIF analyses_._

### Effect of ROI: pixel-by-pixel vs. whole tumor

*k*_ep_ and *v*_p_ values were significantly lower for pixel-by-pixel compared to whole ROI analyses (p<0.0006), and not significantly different for *K* ^trans^ and *v*_*e*_ values (Tables 1 and 2; Fig 3 (1^st^ and 3^rd^ vs. 2^nd^ and 4^th^ columns)).

There were no significant differences in variability (wCV) between pixel and whole ROI analyses across all the DCE-MRI parameters (*K* ^trans^, *k*_ep_, *v*_e_ and *v*_*p*_).

### Between- and within-animal variation

The results of our variance component analyses are presented in Table 3. This shows generally larger intra-rat than inter-rat variances across all DCE-MRI parameters.

**Table 3:** Summary of variance components analysis, by analytical method (n = 12). Model dependent (*K* ^trans^, *k*_ep_, *v*_e_ and *v*_p_).

			Whole ROI		Pixel ROI	
VIF	Parameter model	Parameter	inter-VC	intra-VC	inter-VC	intra-VC
**POP**	**2 para**	***K*** ^**trans**^	0.084	0.208	0.076	0.165
		***k***_**ep**_	0.003	0.018	0.001	0.022
		***v***_**e**_	0.061	0.191	0.062	0.159
**POP**	**3 para**	***K*** ^**trans**^	0.091	0.232	0.083	0.185
		***k***_**ep**_	0.008	0.012	0.004	0.017
		***v***_**e**_	0.058	0.185	0.058	0.176
		***v***_**p**_	0.083	0.186	0.081	0.202
**INDIV**	**2 para**	***K*** ^**trans**^	0.048	0.112	0.041	0.081
		***k***_**ep**_	0.015	0.082	0.005	0.087
		***v***_**e**_	0.012	0.022	0.011	0.024
**INDIV**	**3 para**	***K*** ^**trans**^	0.000	0.110	0.004	0.088
		***k***_**ep**_	0.000	0.097	0.000	0.122
		***v***_**e**_	0.010	0.023	0.011	0.029
		***v***_**p**_	0.142	0.214	0.140	0.327

POP: population averaged VIF

INDIV: individual measured VIF

Inter-VC: between-rat variance component

Intra-VC: within-rat variance component

Intra-VC > inter-VC: indicates that within-rat variation > between-rat variation

n/a: not applicable

## Discussion

The parameters evaluated here are widely regarded by drug developers and others as “biomarkers” within the definition introduced by Atkinson et al. [26]. However, to qualify as biomarkers, they must be “objectively quantified” [26]. If different studies derive different values for the same biomarker because of subtle differences in analysis, the “objective quantification” is deficient [27]. This work was undertaken to assess the impact on parameter values and repeatability of commonly utilized analytical methods in the DCE-MRI arena. Specifically, we explored the effects of two commonly utilized tracer kinetic models (2-parameter *vs*. 3-parameter), two VIF options (population- *vs*. individual-based), and two ROI analytical approaches (whole tumor ROI *vs*. pixel-by-pixel tumor ROI). Each, in principle, has its theoretical advantages and disadvantages.

Our results suggest that DCE-MRI parameter values vary widely depending on the analytical methods utilized, in some cases almost two-fold (*e*.*g*., *K* ^trans^, *k*_ep_, *v*_p_). Overall wCV values also varied widely by analytical methods, with wCVs ranging as follows: *K* ^trans^ 32.9–61.9%; *k*_ep_, 11.6–41.9%; *v*_e_, 16.1–54.9%; and *v*_p_, 53.9–77.2%.

In terms of the tracer kinetic models utilized, the 3-parameter model might in principle be expected to provide a more complete reflection of the underlying tracer kinetics compared to the 2-parameter model since it does not neglect the intravascular tracer contribution (*i*.*e*., the *v*_p_ term) as does the 2-parameter model. Our results suggested that the 3-parameter model yielded significantly lower *K* ^trans^ and *k*_ep_ values than the corresponding 2-parameter model, and no significant differences in *v*_e_ values. Since the 2-parameter model neglects the intravascular signal, the bias (i.e. artefactual elevation of *K* ^trans^ and *k*_ep_ in the 2-parameter model) is not unexpected. Absolute values of *K* ^trans^ and *k*_ep_ from different studies which use these different models cannot be assumed comparable, even though the same biomarker name and units are reported. There were no significant differences in wCVs for all parameters, which suggests that repeatability was not substantially affected by the physiological model applied for these animals.

With regards to the choice of VIF inputs for the model-based analyses, utilization of individual-based VIFs might be expected to yield more reliable results than using a population-based VIF, since vascular tracer profiles can vary quite widely due to variations in IV contrast delivery, cardiac output, renal function, *etc*. It is quite possible, however, that the theoretical advantage of utilizing individual-based VIFs might be out-weighed by the substantial technical challenges in obtaining reliable VIFs from DCE-MRI studies. Our results indicated that there were no significant differences in DCE-MRI parameter values obtained between individual- and population-based VIF analyses. However, wCVs when using individual VIFs were significantly smaller for *v*_e_ than when using the population VIF, and conversely for *k*_ep_. In circumstances in which individually measured VIFs might be unreliable, the utilization of population VIFs may be necessary [19,28]. Our results do not suggest that the use of a population VIF will bias the absolute DCE-MRI biomarker values.

Since each of our DCE-MRI biomarkers is an intensive scalar variable, and since the voxel volumes are identical, the whole-tumor value for each biomarker should in principle be identical to the mean of the individual voxel biomarker values. However, for real-world (noisy) data, that identity may be lost in the propagation of errors, leading to variability and/or bias. Regarding pixel-by-pixel *vs*. whole tumor based ROI evaluations, the former, in principle, is able to display and better reflect the heterogeneous nature of tumors. However, it is computationally more demanding and more vulnerable to signal-to-noise ratio constraints. Furthermore, deriving a simple statistic that adequately summarizes the resultant individual pixel-based parameter distributions is challenging. Adopting the simple approach of using median values to summarize the distributions, our results indicated that pixel-by-pixel based evaluation of ROIs yielded significantly lower *k*_ep_ and *v*_p_ values than whole-tumor based ROI evaluations. As such, it may be important in some circumstances to be able to capture detailed spatial information about tumor heterogeneity.

The tumor time-intensity profile is clearly affected by the tracer input profile. The latter in turn is affected by tracer input delivery (*i*.*e*., the intravenous injection) and physiological parameters (*e*.*g*., cardiac output and renal/excretion function), all of which can vary between studies. Acquisition of a reliable VIF presents substantial challenges. Difficulties include motion- and flow-related artifacts. The difficulties of acquiring reliable VIFs are compounded in small animal studies by the very small cross-sectional area of the major vessels. Indeed, intensity variations (from noise and artifacts) were evident in our VIF time-intensity plots (Fig 1A). We were able to mitigate the flow related artifacts in our acquisition protocol by application of a saturation band between the site of injection and the imaging volume, but inevitably with some loss of temporal resolution. We took precautions to control for the delivery of intravenous contrast medium. We used a pump injector, with fixed gadolinium and saline flush volumes and flow rates, a fixed site of injection (the tail vein), and a constant length of tubing between the injector and tail vein.

There have been some conflicting reports as to the effect of using individual- compared to population-based VIFs: Rijpkema and co-authors [29] has reported that individual arterial input functions (AIFs), compared to population-based AIFs, improved repeatability of *k*_ep_. Parker and co-authors [28] reported that variation in *K* ^trans^, *v*_e_, and *v*_p_ values were smaller when using a population-based AIF compared to an individual-based AIF in a study of tumors in human patients. Their differing conclusions may be partly due to the relative differences in the consistency of the individual VIFs obtained in their studies. Also a variety of models have been proposed to derive population VIFs, and these two studies employed different approaches. The extent to which such models might influence the conclusions is beyond the scope of this work.

The differing views related to VIF estimations in the studies above in humans are paralleled in the pre-clinical arena. The small blood volume and rapid vascular dynamics inherent to small animals necessitate very rapid sampling schemes in order to accurately capture the peak of intravascular enhancement, corresponding to the maximum concentration of contrast agent after injection, and acquisition strategies that are tuned for rapid AIF sampling generally compromise the spatial resolution and coverage of tumor. Studies utilizing acquisitions that are optimized for AIF measurement with very rapid sampling may provide reduced variability using individual measurements [23,30,31]. In the absence of AIF estimates with high temporal resolution, or in the presence of high noise, repeatability may be improved by use of a parameterized population average [19]. It has also been shown that measurements derived from individual and averaged AIFs correlate strongly when a strictly controlled contrast administration protocol is used [20]. In this work, we employed a 3D acquisition protocol that is biased towards anatomic coverage with relatively slow temporal sampling of the AIF. Our study identified no statistically significant differences in parameter values when using the individual or averaged VIFs, but did find some differences in repeatability (wCV) with some specific parameters.

Previous studies with small animals have reported intra-animal wCVs for *K* ^trans^ and *v*_e_ of 18% and 7%, respectively, in a mouse model using PC3 prostate tumors, a 2-parameter model and a pooled (population) VIF [32]. A previous study of ours which compared DCE-MRI and DCE-CT in the same tumor model as in the current study reported wCVs for *K* ^*trans*^, *k*_*ep*_ and *v*_*e*_ of 23%, 16% and 20% respectively [33]. This study employed a different intravenous injection technique, VIF model and acquisition protocol compared to the current study. The first study above examined repeatability over just two, unlike our three, scan visits in our previous and current studies.

Our variance components analysis allowed us to assess the relative contributions of variations between animals (inter-rat) and between individual scan days (intra-rat), to the overall variation. We found that for all DCE-MRI parameters, the intra-rat contributions were comparable to or larger than the inter-rat contributions to the overall variation in this C6 model. Tumor vasculature is intrinsically chaotic and unstable, and it is not surprising that its day-to-day variations may exceed relatively small between-rat variations, given the uniform and controlled tumor implantation techniques and animals utilized.

We acknowledge and recognize limitations in our study. Our study was undertaken with only one tumor model. The physiological compartment models used in our study are widely used. However, we utilized a single software implementation of these models in this study. It is likely that algorithmic differences within software implementations may give rise to differences in the parameter values.

In our repeatability study, it would have been desirable to obtain scan-rescan measurements in close succession. Unfortunately, any persistent gadolinium would potentially alter signal intensities and saturation, thereby confounding the measurements. It was considered that this difficulty would be mitigated by imposing a one day interval between scans. Unlike other studies which have restricted their evaluations to just two sequential evaluations, we undertook serial imaging evaluations on three consecutive days. Tumors inevitably changed in size and potentially in their perfusion properties over the 48 hours of our serial imaging. Although we observed increases in tumor size with time, we did not observe any systematic changes in perfusion parameters on formal statistical testing (data not presented). Even if possible, undertaking repeat scans in short succession in one scanner visit would have removed important variables in DCE-MRI experiments, which include handling of animals, anesthesia and physical re-positioning/localization. The variabilities obtained, therefore, more closely mimic longitudinal experiments in which there are typically intervening therapeutic interventions.

We limited our tumor ROI evaluations to a single observer; an examination of inter- and intra-observer variability was beyond the scope of this work. It was also beyond the scope of the current study to explore the impact of our various analytical options with different animal models and DCE-MRI acquisition protocols.

Our results indicate that DCE-MRI parameter values and repeatability can vary widely depending on the specific analytical methods utilized. Comparisons across studies especially of absolute parameter values should be interpreted with caution. Given the wide ranges in variability of parameters, it would be prudent to incorporate scan-rescan repeatability in studies. Efforts should be made to acquire reliable VIF data for analyses. An understanding of repeatability of DCE-MRI measurements provides insight into what observed changes can be considered significant, and can also assist in design of future studies and sample-size calculations.
